# Chemical Affinity between Tannin Size and Salivary Protein Binding Abilities: Implications for Wine Astringency

**DOI:** 10.1371/journal.pone.0161095

**Published:** 2016-08-12

**Authors:** Wen Ma, Pierre Waffo-Teguo, Michael Jourdes, Hua Li, Pierre-Louis Teissedre

**Affiliations:** 1 College of Enology and Shaanxi Engineering Research Center for Viti-Viniculture, Northwest A & F University, Yangling, Shaanxi, China; 2 Univ. de Bordeaux, ISVV, EA 4577, Unité de recherche OENOLOGIE, F-33882, Villenave d'Ornon, France; 3 INRA, ISVV, USC 1366 OENOLOGIE, F-33882, Villenave d'Ornon, France; Wuhan Botanical Garden, CHINA

## Abstract

Astringency perception, as an essential parameter for high-quality red wine, is principally elicited by condensed tannins in diversified chemical structures. Condensed tannins, which are also known as proanthocyanidins (PAs), belong to the flavonoid class of polyphenols and are incorporated by multiple flavan-3-ols units according to their degree of polymerization (DP). However, the influence of DP size of PAs on astringency perception remains unclear for decades. This controversy was mainly attributed to the lack of efficient strategies to isolate the PAs in non-galloylated forms and with individual degree size from grape/wine. In the present study, the astringency intensity of purified and identified grape oligomeric tannins (DP ranged from 1 to 5) was firstly explored. A novel non-solid phase strategy was used to rapidly exclude the galloylated PAs from the non-galloylated PAs and fractionate the latter according to their DP size. Then, a series of PAs with individual DP size and galloylation were purified by an approach of preparative hydrophilic interaction chromatography. Furthermore, purified compounds were identified by both normal phase HPLC-FLD and reverse phase UHPLC-ESI-Q-TOF. Finally, the contribution of the astringency perception of the individual purified tannins was examined with a salivary protein binding ability test. The results were observed by HPLC-FLD and quantified by changes in PA concentration remaining in the filtrate. In summary, a new approach without a solid stationary phase was developed to isolate PAs according to their DP size. And a positive relationship between the DP of PAs and salivary protein affinity was revealed.

## Introduction

**Fig 1:**
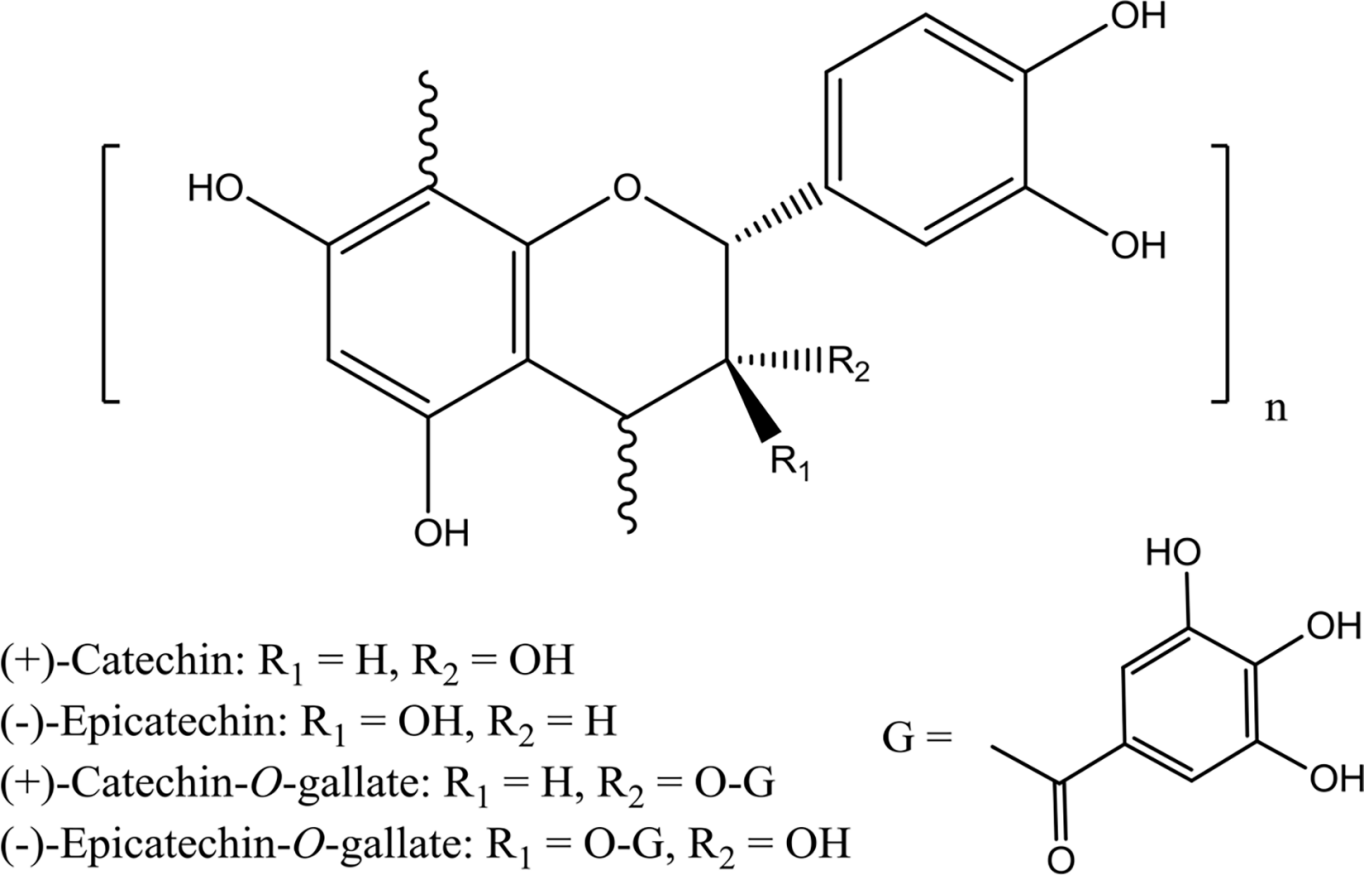
Subunits structures of tannins from grape seed.

Condensed tannins, which are also known as proanthocyanidins (PAs), are oligomers and polymers of flavan-3-ols units belonging to the flavonoid class of polyphenols that are widely distributed throughout the plant kingdom and their derived products. Owing to their considerable contribution to nutritional functions [1–3] and sensory properties [4, 5], PAs have attracted much interest in recent decades [6–9]. In grape seeds and skins, PAs present as a heterogeneous mixture involving various degrees of both polymerization and galloylation (Fig 1), which probably corresponds to distinctive bioactivities [10]. Involving several isomeric flavan-3-ols and their galloylated derivatives, the subunits of grape tannins are more complicated than those of PAs in other plants. Such as in cacao, PAs can be well-separated according to degree of polymerization using a diol stationary phase of HPLC [11]. Given the lack of efficient purification methodologies, few PAs derived from grape with individual high DP size have ever been either purified or studied. Therefore, quantification of the large PAs in grape and wine remains problematic and their bioactivities are barely understood. Knowledge regarding condensed tannins in wine remains limited to the several well-known tannin molecules.

In recent decades, numerous attempts have been made to isolate PAs from grape or wine. Although gel permeation chromatography approaches (such as Sephadex LH-20, TSK, PLgel and C18 Sep-Pak cartridges) [12–21] or preparative normal phase HPLC [11] have been used to attempt separation, obvious drawbacks have hampered the progress of these solid-phase chromatographic approaches. For example, the resolution of the gel permeation chromatography was too low to discriminate the extent of individual DP sizes, and preparative normal phase HPLC proved costly and ill-suited to conduct a study on a preparative scale. Furthermore, the traditional methods were tedious, time-consuming and results were not satisfactory.

Astringency, an essential parameter for high-quality red wine, is an oral sensation involving dryness and puckering. So far, it is generally thought that the perception of astringency in wine is primarily due to condensed tannins derived from grape, their mechanism being principally explained by non-covalent interactions between condensed tannins and salivary protein [4]. Many factors impact the intensity of the perception of wine astringency. However, controversy concerning the influence of DP size of PAs on astringency perception has existed for decades. Some believe that astringency intensity increases with DP size [22], whereas others argue that there is an inflexion point in this parabolic trend [23]. Nevertheless, all studies to date on PAs astringency level have been performed PAs with the average DP and without elimination of the galloylated tannins, rather than on purified fraction with specific DP and without galloylated tannins.

**Fig 2:**
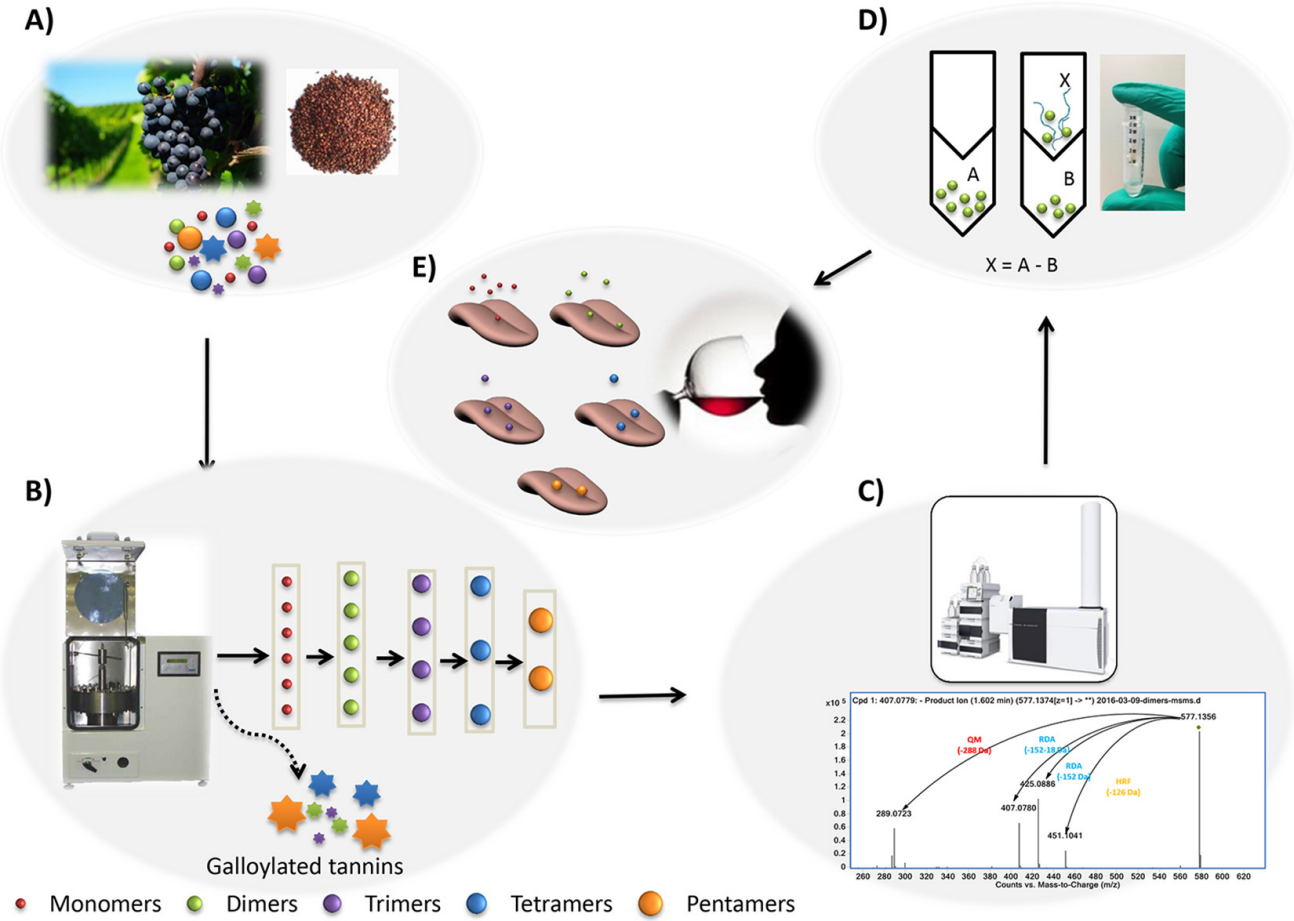
General procedure of experimental design: (A). Tannins were extracted from grape seeds; (B). Technique of CPC fractionated tannins according to their DP size; (C). Purified compounds were identified by UHPLC-HRMS; (D). Salivary protein binding abilities of purified oligomers were assessed by measuring difference between tannins with and without saliva; (E). Astringency intensities of monomeric, dimeric, trimeric, tetrameric and pentameric tannins in model wine solution were estimated.

The aims of this study was to develop an efficient strategy to isolate tannins both in non-galloylated forms and at individual DP and to reveal the chemical affinity between tannin DP and salivary protein binding abilities, which implicates for wine astringency. The experiment design is as shown in Fig 2.

## Materials and Methods

### Study design

The authority who issued the permission for the grapes collection 2014 for the study is Union des Producteurs Saint Emilion in France.

Internal committee for ethics of Laboratory Research Unit USC 1366 Board, Institut des Sciences de la Vigne et du Vin of Bordeaux University approved the study for saliva collection of volunteers. All partcipants sign up an inform consent form with type of research, voluntary participation and saliva collection protocol by spitting, with confidentiality."

### Chemicals and samples

Deionized water was purified with a Milli-Q water system (Millipore. Bedford. MA. USA). Acetonitrile (HPLC grade), methanol (HPLC grade), glacial acetic acid (analytical grade), chloroform, ethyl acetate, ethanol and acetone were purchased from Prolabo-VWR (Fontenays/Bois. France). Trifluoroacetic acid was bought from Sigma-Aldrich. Water (Optimal® LC/MS), MeOH (Optimal® LC/MS) and formic acid (Optimal® LC/MS) were obtained from Fisher Scientific (Geel. Belgium) for UHPLC-Q-TOF analysis.

The grape variety was *Vitis vinifera* L. cv. Cabernet Sauvignon from the vineyard of appellation Saint-Emilion located in the Bordeaux vine growing region in the southwest of France.

### Grape seed procyanidins extraction

Grape seeds were removed by hand from grapes, lyophilized for 2 days and stored at -20°C. The frozen seeds were finally ground in a ball grinder. An ASE 350 Accelerated Solvent Extraction System (Dionex Corporation. Sunnyvale. CA) was used as previously [24] to extract the tannins from the ground seeds. The ground grape seed and skin (~10 g) were submitted to eight solid/liquid consecutive extractions with acetone/water (80:20, v/v) as solvent systems (40 mL of the corresponding solvent system). The ASE experimental variables were pressure (1500 psi), temperature (40°C), static time (4 min), and preheat time (5 min), by using an N_2_ flush to prevent oxidation during extraction. The volume of all collection tubes was combined after extraction and then evaporated under reduced pressure. The obtained solid residue was re-dissolved in 30 mL of water and lyophilized.

The extract was solubilized in 250 mL of water/ethanol (95:5, v/v) and extracted three times with chloroform (v = 250 mL) to remove lipophilic material. Then the aqueous phase was extracted three times with ethyl acetate (v = 250 mL) to obtain two distinctive fractions [25]. The organic fraction was concentrated and lyophilized to obtain a dry powder. A crude oligomeric PAs extract was obtained.

### Non-solid phase fraction

The centrifugal partition chromatography (CPC) apparatus was an FCPC 1000 provided by Kromaton Technologies (Saintes-Gemmes-sur-Loire, France). It consisted of a rotor (45 circular partition disks; total column capacity of 940 mL; 1440 partition cells), a binary high-pressure gradient pump (Gilson 321-H1), a high pressure injection valve (50 mL sample loop, Rheodyne) and a Kromaton UV–vis detector. Fractions were collected by an Advantec CHF 122SC fraction collector. PAs were separated by a two-phase system ethyl acetate-ethanol-water (6:1:5, v/v/v). For each injection, 5 g of extract were dissolved in 10 mL of the upper and lower phases (50/50, v/v) of the system and 0.45 mm filtered. Experiments were carried out in ascending mode at 1000 rpm with a flow rate of 15 mL/min for 140 min. The fraction collector was set to 1 tube/min. Every five CPC tubes, an aliquot (200 μL) was taken, evaporated, dissolved in 1 mL of H_2_O/MeOH (50:50, v/v) and analyzed by UHPLC-ESI-Q-TOF. When grouping the tubes, samples presenting the similar HPLC profiles were pooled together, evaporated *in vacuo*, suspended in water and freeze-dried. Five determined fractions were obtained.

### Preparative High Performance Liquid Chromatography Purification

Purification was performed on a Luna HILIC column (21.2 × 250 mm, 5 μm, Phenomenex) by a Varian LC machine consisting of a Prostar 210 two-way binary high-pressure gradient pump, a 2 mL loop and a Prostar 325 UV/Visible detector. Chromatographic peaks were manually collected. The mobile phase consisted of acidified acetonitrile (Eluent A) and acidified aqueous methanol (Eluent B. Methanol: water. 95:5. v/v), both containing 0.025% trifluoroacetic acid. The flow rate was 22 mL/min and eluent B followed this gradient: 0 min, 7%; 57 min, 37.6%; 60 min, 100%; 67 min, 100%; 73 min, 7%; 83 min, 7%, 52 min. For each injection, 100 mg of fraction compounds were dissolved in 0.5 mL methanol and manually injected into the system. UV detection was carried out at 254 nm and 280 nm. After successive injections, the purified oligomers were evaporated *in vacuo* to remove solvent and freeze-dried to obtain the targeted monomers, dimers, trimers, tetramers and pentamers in powder.

### UHPLC-HRMS in reverse phase analysis

The UHPLC-HRMS system used was an Agilent 1290 Infinity equipped an ESI-Q-TOF mass spectrometer (Agilent 6530 Accurate Mass). The UHPLC-HRMS analyses were carried out on a C18 UHPLC column (2.1 × 100 mm, 1.8 μm, Agilent). The mobile phases were water (Eluent A) and acetonitrile (Eluent B), both containing 0.1% formic acid. For monomers, dimers, trimers and tetramers, the gradient of solvent B was as follows: 3% for 0.8 min; 3 to 5% for 2 min; 5% for 1.4 min; 5 to 7% for 1.4 min; 7 to 10% for 0.5 min; 10% for 1.4 min; 10 to 12% for 1.4 min; 12 to 14% for 0.5 min; 14 to 25% for 3 min; 25 to 100% for 0.3 min and 100% for 2.3 min. For the pentamers, the gradient of solvent B was 15% for 5 min; 15% to 25% for 10 min; 25% to 100% for 2 min; 100% for 3 min. The UHPLC column was equilibrated for 3 min using the initial condition before the next injection. This UHPLC system was coupled to an ESI-Q-TOF-MS with an electrospray ion source with Agilent Jet Stream Technology. The mass spectrometer was operated in extended dynamic range of 2 GHz (m/z 3200 Th). The nebulizer pressure and flow rate were set at 25 psi and 9 L/min, respectively. Its drying gas temperature was 300°C. The sheath gas flow and temperature were set at 11 L/min and 350°C. The fragmentation, skimmer, OCT and capillary voltage were at 150 V, 65 V, 750 V and 4000 V, respectively. All the analyses were performed in negative mode. The collision energies used for MS/MS analysis were 10 V, 15 V or 30 V for different compounds. The data analysis was performed on Mass Hunter Qualitative Analysis software.

### HPLC-FLD in normal phase analysis

A Thermo-Finnigan Surveyor system was used for the normal phase HPLC analysis, consisting of a quaternary pump (Surveyor LC pump Plus), an autosampler (Surveyor autosampler Plus), a UV–vis detector (Surveyor PDA Plus) and a fluorescence detector (Surveyor FL Plus Detector). This HPLC-UV system was also coupled to a Thermo-Finnigan LCQ Advantage spectrometer equipped with an electrospray ionization source and an ion trap mass analyzer. Fluorescence and mass data were analyzed by ChromQuest 4.2 and Xcalibur 2.2.0 software, respectively.

Separation was performed on normal phase Luna HILIC column (4.6 × 250 mm, 5 μm, Phenomenex). The separation condition was reported previously [11]. Briefly, the mobile phase consisted of acidified acetonitrile (Eluent A, acetonitrile: acetic acid, 98:2, v/v) and acidified aqueous methanol (Eluent B, methanol: water: acetic acid, 95:3:2, v/v/v). The flow rate was 1 mL/min and eluent B followed this gradient: 0 min, 7%; 57 min, 37.6%; 60 min, 100%; 67 min, 100%; 73 min, 7%; 83 min, 7%, 52 min. Calibration curves were established with excitation at 276 nm and emission at 316 nm using external purified standards (PAs monomers, dimers, trimers, tetramers and pentamers). Each sample was injected three times. Unknown concentrations were determined from the regression equations.

### Saliva binding ability test

A pool of saliva was collected from 20 volunteers (10 males and 10 females aged 20 to 35 years old). They were in good health and not undergoing oral treatment. They were previously instructed to avoid smoking on the saliva donation day and take no food or beverages for at least 1 h before collection. Collection time was standardized between 10–12 a.m. to reduce the concentration variability. Saliva was collected with no oral stimulus but rather with a visual stimulus by lemons. The saliva was collected by small bottles and immediately frozen at -20°C after collection. After all the samples had been collected, they were thawed, pooled and refrozen for lyophilization to concentrate the nature saliva around three-fold. After lyophilized concentration, the thawed saliva sample was centrifuged at 8,000 g for 5 min at 4°C by a Jouan MR22 refrigerated centrifuge. The supernatants were the targeted saliva protein sample.

The method was that of Schwarze and Hofman [26] with some modifications. Purified tannins (monomers, dimers, trimers, tetramers and pentamers) were prepared as a dissolution at the concentration of 1.5 mg/mL in model wine solution (ethanol 12%; tartaric acid = 1 g/L; pH = 3.5). Tannin solution (300 μL) was mixed with 700 μL of prepared saliva sample or water (as control) and incubated at 37°C for 5 min. After incubation, an aliquot (400 μL) of the mixture was moved to a 3k Da centrifugal filter (Amicon Ultra-0.5 Centrifugal Filter 3k Devices, Merck Millipore) and centrifuged at 18,000 rpm for 5 min at 37°C. The filtrate in the bottom, namely the non-bound tannins, was injected into the normal phase HPLC-FLD for quantitative analysis. Each analysis was performed in duplicate.

### Data analysis

The amount of interacted tannins (X) was calculated as the difference in tannin concentration in solution with (B) and without salivary protein (A): X = A–B. Statistical data analysis was performed using the analysis of variance (ANOVA) of Statistica V.7 software. Tukey’s HSD and Duncan’s tests were used as comparison tests when samples were significantly different after ANOVA (p < 0.05)

## Results and Discussions

### Isolation of grape oligomeric tannins in individual DP size

**Fig 3:**
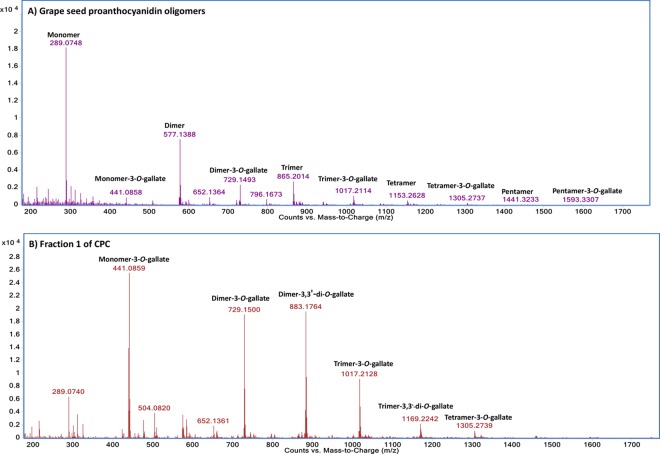
MS spectra obtained for crude extract (A) and fraction 1 of CPC (B).

In the crude grape seed PA oligomer extract, PAs and their galloylated derivatives were found as a mix (Fig 3A). After CPC had run in ascending mode for 140 mins, six fractions were produced (Table 1). The first (F1, 1.72 g, 40%) was comprised of multiple galloylated PAs (as shown in Fig 3B). The main products of fraction two (F2, 1.04 g, 21.8%), fraction three (F3, 546.9 mg, 11.41%), fraction four (F4, 302.3 mg, 6.31%) and fraction five (F5, 257.2mg, 5.36%) corresponded to monomers, dimers, trimers and tetramers/pentamers, respectively (S1 Fig). The application of CPC on grape PAs fractionation was less-time consuming, gave a high recovery, could potentially be scaled-up and was less expensive thanks to low solvent costs and the absence of expensive adsorbents. In general, the total recovery yield of CPC was 87.74%, which was much higher than any traditional solid-phase separation strategies to date. Specifically, the untargeted compounds (galloylated PAs) with a high percentage were excluded in the very beginning, while the targeted PAs compounds were fractionated consecutively according to their DP. The mass response of dimers remained high in F4 and F5 owing to the huge quantity of dimers in the crude extract and the weak mass signals of the large PA molecules. Tetramers and pentamers were already present in the crude extract and were enriched in F5 after CPC. Thanks to the enrichment of tetramers and pentamers, the fifth fraction was well-prepared to be purified by a solid-phase chromatography with a low and efficient injection amount in the next step. PAs were purified from 4.8 g of oligomeric PA grape seed extract by the combination of CPC using a ternary biphasic system EtOAc/EtOH/H_2_O (6:1:5, v/v/v) and preparative normal phase HPLC. Although weak signals of hexamers and higher molecules could be detected by HRMS in the tail fraction, their quantities were too low to be isolated or to be used for salivary protein investigation.

**Table 1:** Information on CPC fractions.

Fraction	Main Compounds	Retention time (min)	Weight (mg)	Yield
F1	Galloylated tannins	0–8	1719.6	35.87%
F2	Monomers	9–20	1044.4	21.78%
F3	Dimers	21–30	546.9	11.41%
F4	Trimers	31–42	302.3	6.31%
F5	Tetramers. Pentamers	43–62	257.2	5.36%
F6	Tail fraction	63–140	198.6	4.14%
F0	Coil fraction		172.7	3.6%
Sum			4241.7	87.74%

Individual oligomeric PAs were purified by preparative normal phase HPLC on a Luna HILIC column according to the HPLC gradient of Kelm [11] with the methods transformation. The PAs present as a heterogeneous mixture in grapes, which involves various isomers and are hardly available as pure compounds but rather as a mix [27]. Hence, the attempts to isolate each individual pure PAs molecules with high DP were time-consuming and not really necessary. Therefore, the normal phase column was used to isolate and identify oligomeric PAs according to DP in preparative HPLC-UV and analytical HPLC-FLD, respectively (Extracted ion chromatograms can be found in S2 Fig).

To our knowledge, this is the first time that PAs from grape have been purified in individual DP size up to five. To sum up, the combination of CPC and preparative HPLC allowed the purification of grape PAs in individual DP size.

### Compounds identification by both HPLC-FLD and UHPLC-HRMS

**Fig 4:**
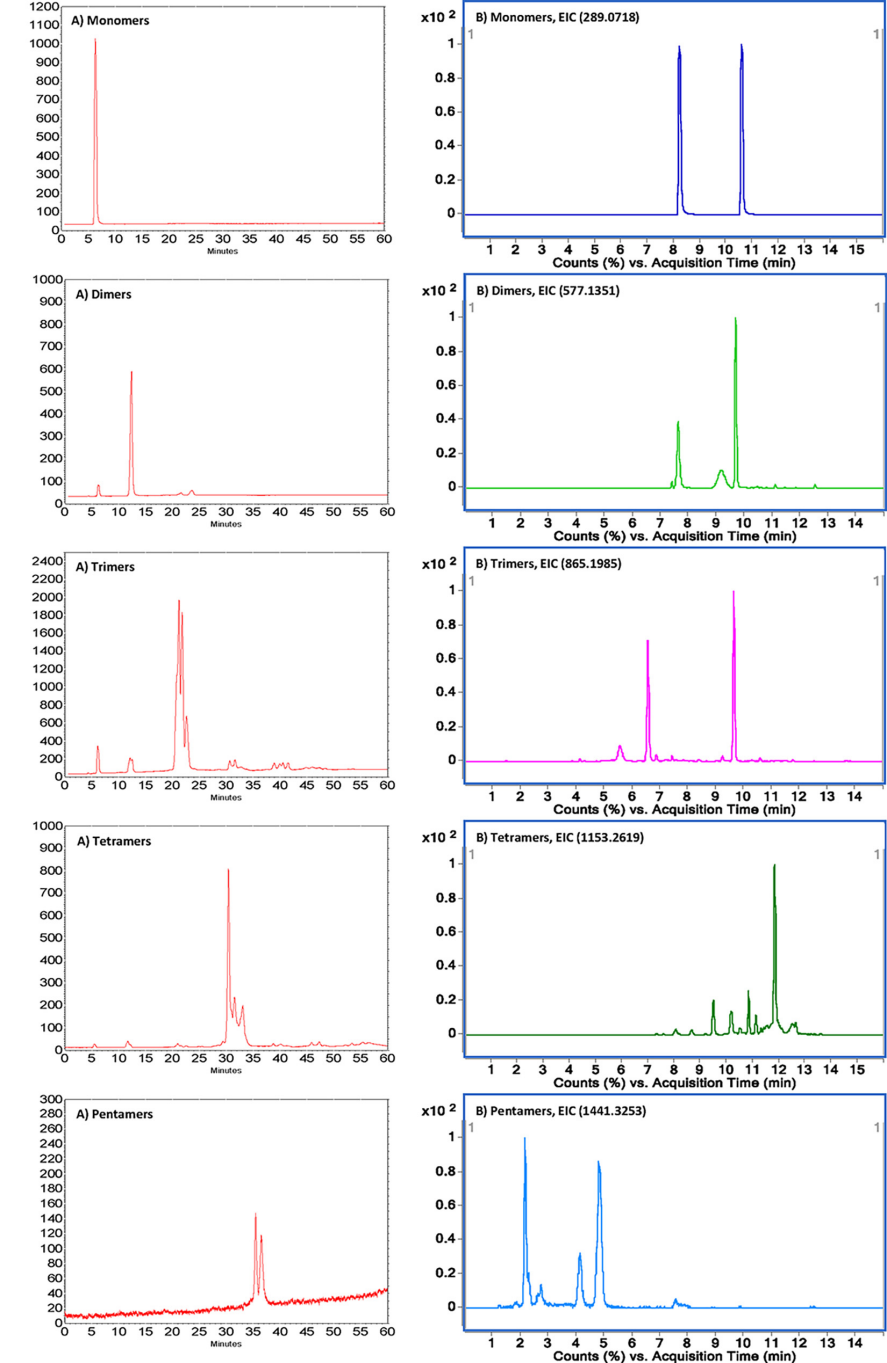
Two complementary and orthogonal HPLC approaches to identify the five purified PAs: A). Normal phase HPLC-FLD chromatograms to separate PAs according to their DP; B). Reverse phase UHPLC-Q-TOF extracted ion chromatograms to separate isomeric PAs at the same DP.

Identification of the purified compounds is illustrated in Fig 4 by two complementary and orthogonal approaches: the normal phase HPLC-FLD system and a reverse phase UHPLC-Q-TOF system. A series of PAs with individual DP were obtained: monomers (white powder; purity: 99.9%), dimers (white powder; purity: 87.7%), trimers (light yellow powder; purity: 83.2%), tetramers (light yellow powder; purity: 92.5%) and pentamers (light yellow powder; purity: 99.9%). The purity was examined by HPLC-FLD (Fig 4A) and the fluorescence absorbance declined with the rise in DP [28]. As shown in Fig 4B, MS identification of compounds was performed with UHPLC-HRMS equipment in reverse phase. Two isomeric monomers, four isomeric dimers, three isomeric trimers, five isomeric tetramers and five isomeric pentamers were found and identified. The detailed high-resolution mass spectrometry data was listed in Table 2.

**Table 2:** HRMS identification data of purified tannins.

No.	Compounds	Rt (min)	Formula [M-H]^-^	Calculated *m/z*	Measured *m/z*	Diff (ppm)
1	Monomer 1	8.20	[C_15_H_14_O_6_-H]^-^	289.0718	289.0718	0
2	Monomer 2	10.57	[C_15_H_14_O_6_-H]^-^	289.0718	289.0716	-0.69
3	Dimer 1	7.39	[C_30_H_26_O_12_-H]^-^	577.1351	577.1341	-1.73
4	Dimer 2	7.61	[C_30_H_26_O_12_-H]^-^	577.1351	577.1340	-1.91
5	Dimer 3	9.15	[C_30_H_26_O_12_-H]^-^	577.1351	577.1342	-1.56
6	Dimer 4	9.66	[C_30_H_26_O_12_-H]^-^	577.1351	577. 1343	-1.39
7	Trimer 1	5.55	[C_45_H_38_O_18_-H]^-^	865.1985	865.1977	-0.92
8	Trimer 2	6.55	[C_45_H_38_O_18_-H]^-^	865.1985	865.1985	0.00
9	Trimer 3	9.63	[C_45_H_38_O_18_-H]^-^	865.1985	865.1997	1.39
10	Tetramer 1	9.47	[C_60_H_50_O_24_-H]^-^	1153.2619	1153.2589	-2.6
11	Tetramer 2	10.15	[C_60_H_50_O_24_-H]^-^	1153.2619	1153.2586	-2.86
12	Tetramer 3	10.82	[C_60_H_50_O_24_-H]^-^	1153.2619	1153.2596	-1.99
13	Tetramer 4	11.10	[C_60_H_50_O_24_-H]^-^	1153.2619	1153.2584	-3.03
14	Tetramer 5	11.81	[C_60_H_50_O_24_-H]^-^	1153.2619	1153.2611	-0.69
15	Pentamer 1	2.17	[C_75_H_62_O_30_-H]^-^	1441.3253	1441.3248	-0.35
16	Pentamer 2	2.29	[C_75_H_62_O_30_-H]^-^	1441.3253	1441.3239	-0.97
17	Pentamer 3	2.70	[C_75_H_62_O_30_-H]^-^	1441.3253	1441.3258	0.35
18	Pentamer 4	4.13	[C_75_H_62_O_30_-H]^-^	1441.3253	1441.3201	-3.61
19	Pentamer 5	4.82	[C_75_H_62_O_30_-H]^-^	1441.3253	1441.3254	0.07

**Fig 5:**
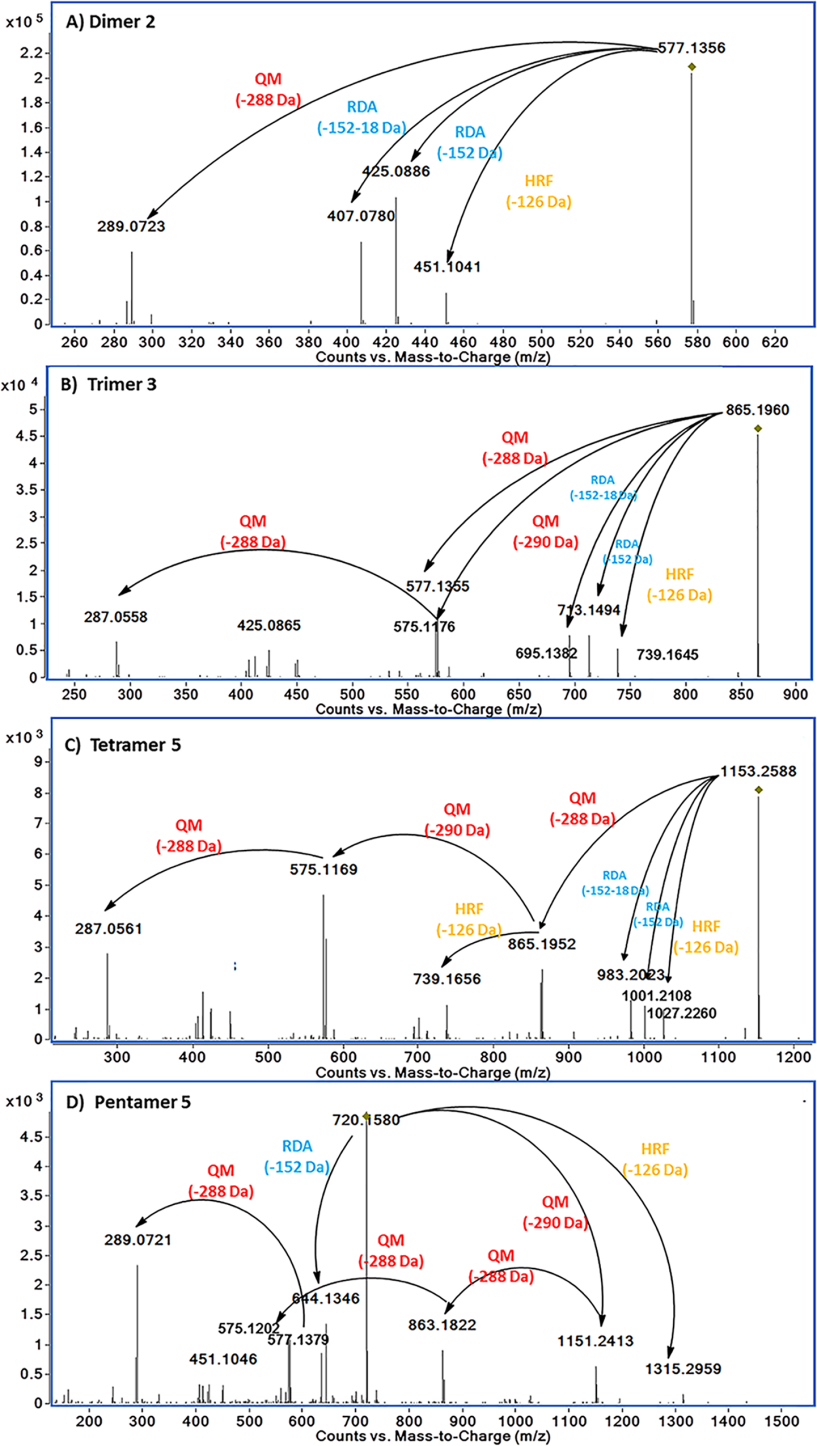
Examples of MS/MS fragments of purified dimeric, trimeric, tetrameric and pentameric PA.

As demonstrated in Fig 5, the main fragmentation pathways of purified PAs in negative ion mode ESI-MSMS spectra were postulated based on the principles of quinone methide fission (QM) with the ion lost from upper unit (-288 Da) and ion lost from lower unit (-290 Da), retro-Diels–Alder fission (RDA, -152 Da) and a loss of water molecule (-18 Da) [29, 30]. Dimer 2 ([M-H]^-^, *m/z* 577.1356, Fig 5A) was diagnosed by the fragment ions with *m/z* 451.1041, 425.0886, 407.0780, 289.0723. The ion with *m/z* 289.0723 ([M-H-QM (288 Da)]^-^) was likely produced after QM cleavage of the [M-H]^-^ ion with the loss of upper unit (epi)catechin. The ions with *m/z* 451.1041, 425.0886, 407.0780 were corresponding to the fissions of HRF (-126 Da), RDA (-152 Da) and RDA+H_2_O (-152–18 Da), respectively. Similarly, after QM cleavage, trimer 3 ([M-H]^-^, *m/z* 865.1960, Fig 5B) was fragmented into 577.1355 ([M-H-QM (288 Da)]^-^) and 575.1176 ([M-H-QM (289 Da)]^-^), with the loss of upper and lower unit, respectively. The fragments with *m/z* 287.0558 could be diagnosed by second fragmentation of either one upper unit loss from the *m/z* 575.1196 or one lower unit loss from the *m/z* 577.1355. The ions with *m/z* 739.1645, 713.1494, 695.1382 were corresponding to the fissions of HRF (-126 Da), RDA (-152 Da) and RDA+H_2_O (-152–18 Da) from the precursor ions (*m/z* 865.1960), respectively.

In Fig 5C, tetramer 5 was identified by the precursor ions ([M-H]^-^, *m/z* 1153.2588) and its fragment ions. Sequencing of this tetramer by QM fissions was straightforward, diagnosed by the fragment ions with m/z 865.1952, 575.1169, 287.0561. The ion with *m/z* 1027.2260 and 739.1656 could be formed via a HRF fission from the precursor ion (*m/z* 1153.2588) and one upper unit cleavage ion (*m/z* 865.1952), respectively. The *m/z* 1001.2108 ion can result from an RDA of ring C of the precursor ions. The ion with *m/z* 983.2023 was derived from both RDA fission and loss of the equivalent of water (18 Da).

In Fig 5D, the precursor ion ([M-2H]^2-^, *m/z* of 720.1580) was cleaved by QM fission into the ions with *m/z* 1151.2413, 863.1822, 575.1202, 289.0721 with the loss of the first, the second, the third and the forth units, respectively. The ion *m/z* 644.1346 was identified as a [M-2H]^2-^ ion after an RDA fission while the ion *m/z* 1315.2959 was observed as a [M-H]^-^ ion after HRF fission from the precursor ion. Hence, this compounds were diagnosed as (epi)catechin-(epi)catechin-(epi)catechin-(epi)catechin-(epi)catechin.

### Relationship between tannin size and salivary protein binding abilities

**Fig 6:**
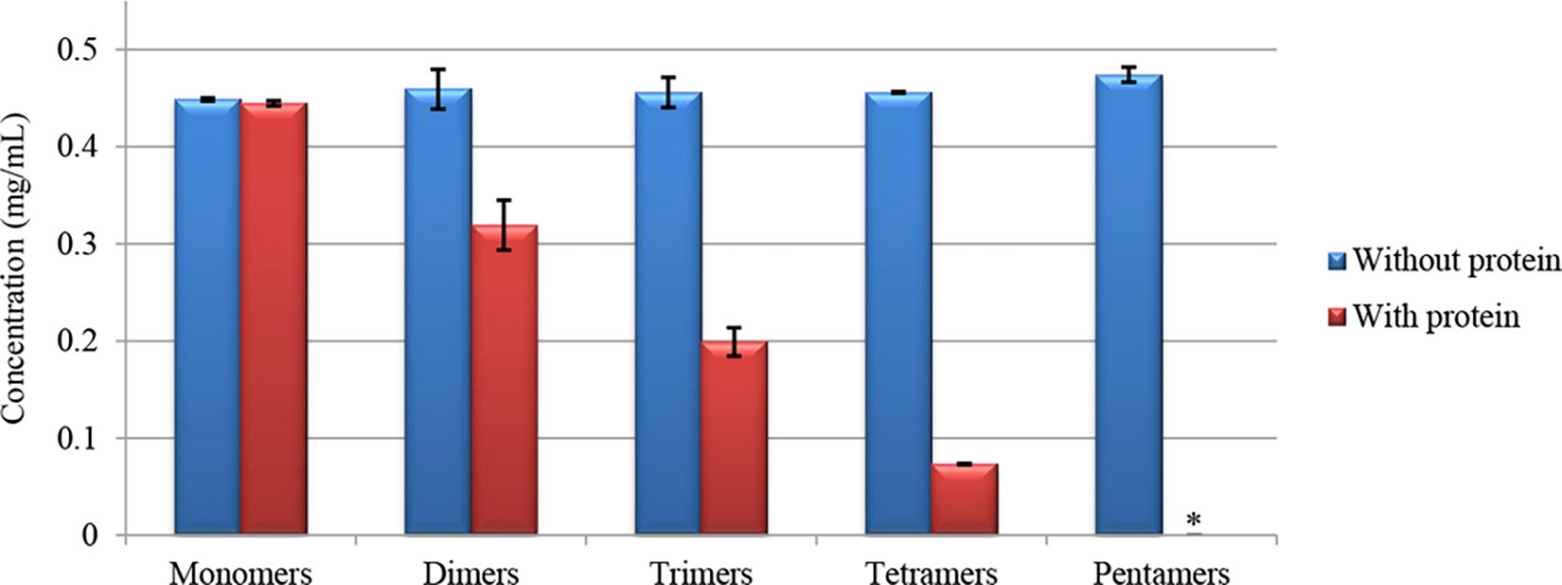
Concentration of oligomeric tannins in filtrates with/without salivary protein interaction (* indicates “not detected”).

The harmony of high-quality red wine is mainly due to the balance of multiple flavors attributed to the numerous chemical components it contains [31]. Tannins are generally believed to interpret the axis of astringency perception. In this investigation, the astringency intensities of the purified PAs were examined by their ability to bind salivary protein and were quantified by HPLC-FLD [26]. A detector of FLD rather than UV was used in order to avoid the UV response of salivary protein at sizes below 3k Da. The amount of interacting tannins was calculated as the difference in tannin concentration in filtrate solution with and without salivary protein. As demonstrated in Fig 6, an obvious decline in interacting tannin concentration from “with” to “without” saliva protein was observed for all of the five PAs. The monomers descended a little whereas there as a remarkable decrease (0.14 mg/mL) in the dimers. More than half of the trimers (0.25 mg/mL) were bound to salivary protein and even more tetramers (0.38 mg/mL) were bound. On the other hand, no pentamers were detected in the filtrate of the sample with salivary protein, indicating that the latter aggregated all the pentamers used in the test. This indicates a positive relationship between DP (ranged from one to five) and the salivary protein affinity of tannins.

This result was in agreement with both the NMR interpretation of saliva protein binding ability to 4 procyanidin dimers (B1-4) and one trimer (C2) [32] and the previous astringency sensory studies on flavan-3-ols monomers, dimers and trimers chemically synthesized [33]. Unfortunately, we could not verify the inflexion point of DP, which was supposed previously [23], the isolation and identification of the grape tannins with higher DP are in need.

## Conclusion

A simple rapid non-solid phase strategy has been developed to efficiently isolate PAs according to DP and to exclude the galloylated PAs. Monomeric, dimeric, trimeric, tetrameric and pentameric PAs were first purified from grape, thereby providing more substances for PA quantification in grape/wine and for further investigations concerning their bioactivity. Furthermore, a tentative test on salivary protein-binding capability was conducted to explore their astringency-stimulating abilities. This is the first report of the astringency activities of identified grape tannins without galloylated forms and in specific DP up to five. The findings of this investigation suggest that the capability of large tannin oligomers to be bound to salivary protein is much stronger than we estimated. Future research focusing on the bioactivities of large PAs molecular are now required.

## Supporting Information

S1 Fig MS spectra obtained for F2, F3, F4 and F5 of CPC (*m/z = [2M-H]^-^; **m/z = [M-2H]^2-^). (EPS)

S2 Fig Extracted ion chromatograms of monomeric, monomer-*O*-gallate, dimeric, trimeric, tetramic and pentameric tannins in normal phase HPLC. (EPS)
